# Steady-state activation of the high-affinity isoform of the α4β2δ GABA_A_ receptor

**DOI:** 10.1038/s41598-019-52573-z

**Published:** 2019-11-05

**Authors:** Spencer R. Pierce, Thomas C. Senneff, Allison L. Germann, Gustav Akk

**Affiliations:** 1 Department of Anesthesiology, Washington University School of Medicine, St. Louis, MO 63110 USA; 2 The Taylor Family Institute for Innovative Psychiatric Research, Washington University School of Medicine, St. Louis, MO 63110 USA

**Keywords:** Ion channels in the nervous system, Molecular neuroscience

## Abstract

Activation of GABA_A_ receptors consisting of α4, β2 (or β3), and δ subunits is a major contributor to tonic inhibition in several brain regions. The goal of this study was to analyze the function of the α4β2δ receptor in the presence of GABA and other endogenous and clinical activators and modulators under steady-state conditions. We show that the receptor has a high constitutive open probability (~0.1), but is only weakly activated by GABA that has a maximal peak open probability (P_Open,peak_) of 0.4, taurine (maximal P_Open,peak_ = 0.4), or the endogenous steroid allopregnanolone (maximal P_Open,peak_ = 0.2). The intravenous anesthetic propofol is a full agonist (maximal P_Open,peak_ = 0.99). Analysis of currents using a cyclic three-state Resting-Active-Desensitized model indicates that the maximal steady-state open probability of the α4β2δ receptor is ~0.45. Steady-state open probability in the presence of combinations of GABA, taurine, propofol, allopregnanolone and/or the inhibitory steroid pregnenolone sulfate closely matched predicted open probability calculated assuming energetic additivity. The results suggest that the receptor is active in the presence of physiological concentrations of GABA and taurine, but, surprisingly, that receptor activity is only weakly potentiated by propofol.

## Introduction

Activation of the Cl^−^ permeable GABA_A_ receptor contributes to cellular inhibition. The two principal types of the GABA_A_ receptor in the central nervous system are the synaptic receptor that is activated phasically by presynaptically released GABA, and the extrasynaptic receptor that is activated tonically by ambient GABA. Native GABA_A_ receptors in the brain are additionally exposed to a number of endogenous GABAergic agents including taurine (2-aminoethanesulfonic acid) and potentiating and inhibitory neurosteroids, that can amplify or inhibit the response to the transmitter. Furthermore, both the synaptic and extrasynaptic GABA_A_ receptors are activated and modulated by clinically used GABAergic sedatives and anesthetics such as propofol and etomidate^1,2^. The two types of receptors differ in their subunit composition; synaptic receptors comprise α1-3, β2-3, and γ2 subunits, whereas extrasynaptic receptors typically consist of α4, β2-3, and δ subunits.

With few exceptions^3,4^, previous functional studies of the α4βδ receptor have concentrated on recording peak current responses, i.e., maximal responses to short-duration applications of one or more agonists. It may be argued that this approach does not accurately reflect native conditions, which can be characterized as essentially infinite-duration exposure to a low concentration of GABA with slowly developing changes in the concentrations of other endogenous agonists and modulators and, if so administered, GABAergic clinical agents. This discrepancy between typical experimental and the presumed *in vivo* conditions makes prediction of normal behavior of the native extrasynaptic receptor and properties of tonic inhibition challenging.

**Figure 1:**
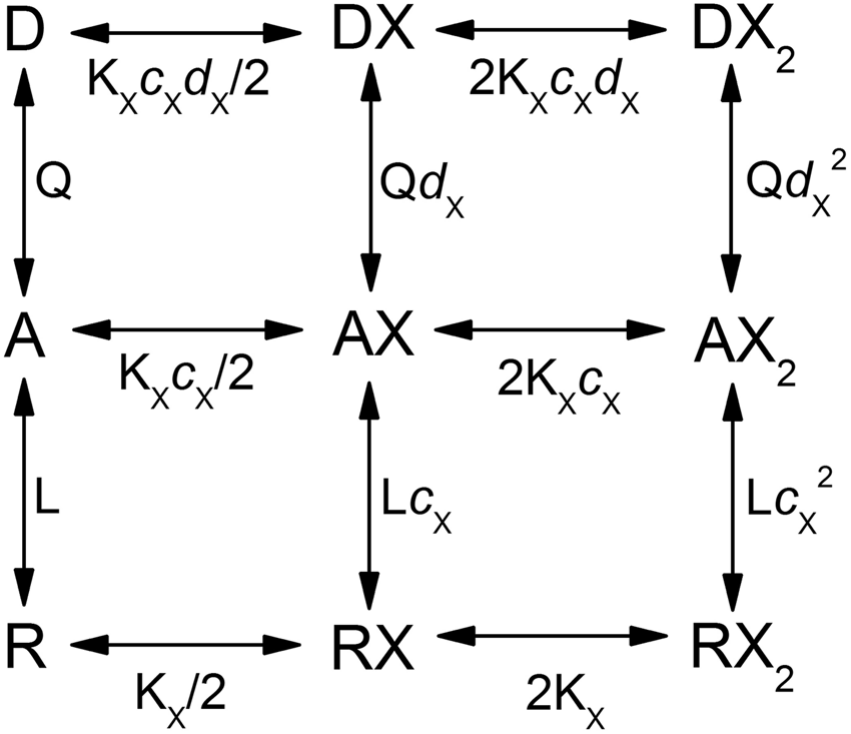
The Resting-Active-Desensitized (RAD) model. The model is shown for agonist X (e.g., GABA) that has two binding sites on the receptor. The receptor can occupy a resting (R), active (A), or desensitized (D) state. The resting and desensitized states are non-conducting, and the active state is conducting (also called “open”). The active and desensitized states have higher affinity to the agonist than the resting state. The parameter L (=R/A) describes the equilibrium between the resting and active states, and the parameter Q (=A/D) describes the equilibrium between the active and desensitized states. K_X_ is the equilibrium dissociation constant for agonist X of the resting receptor. K_X_*c*_X_ is the equilibrium dissociation constant for agonist X of the active receptor, and K_X_*c*_X_*d*_X_ is the equilibrium dissociation constant for X of the desensitized receptor. The inhibitory steroid PS has high affinity to the desensitized state and low affinity to the resting and active states. For the agonists studied, the ratio of affinities for the active and desensitized states (*d*) is close to 1 (see text for additional discussion). The behavior of the receptor in the RAD model is decribed by Eqs (1–3).

We recently described derivation and properties of a three-state Resting-Active-Desensitized (“RAD”) model^5^. The model (Fig. 1), which was initially employed to quantitatively describe steady-state activity in the synaptic-type α1β2γ2L GABA_A_ receptor activated by a single agonist, could also be used to accurately predict steady-state activity in the presence of multiple potentiating and inhibitory agents. Here, we have employed the RAD model to investigate the properties of the human α4β2δ expressed in *Xenopus* oocytes. A major goal of the study was to elucidate steady-state activity in the presence of multiple endogenous and clinical activating (GABA, taurine, propofol, allopregnanolone) and inhibitory (pregnenolone sulfate) agents to predict the behavior of the extrasynaptic GABA_A_ receptor under conditions mimicking the native pharmacological environment.

We show that the receptor has a constitutive open probability of ~0.1 and a steady-state open probability (P_Open,S.S._) near 0.3 in the presence of saturating GABA. The receptor is potently activated by the transmitter GABA, the orthosteric agonist taurine, and the allosteric agonists propofol and allopregnanolone (3α5αP). An agreement between the P_Open,S.S._ calculated using equations derived from the RAD model and the P_Open,S.S._ observed experimentally upon coapplication of combinations of GABA, taurine, propofol, 3α5αP, and the inhibitory steroid pregnenolone sulfate (PS) indicates that the drugs act energetically additively.

## Results

### Activation and desensitization by the orthosteric agonists GABA and taurine

**Figure 2:**
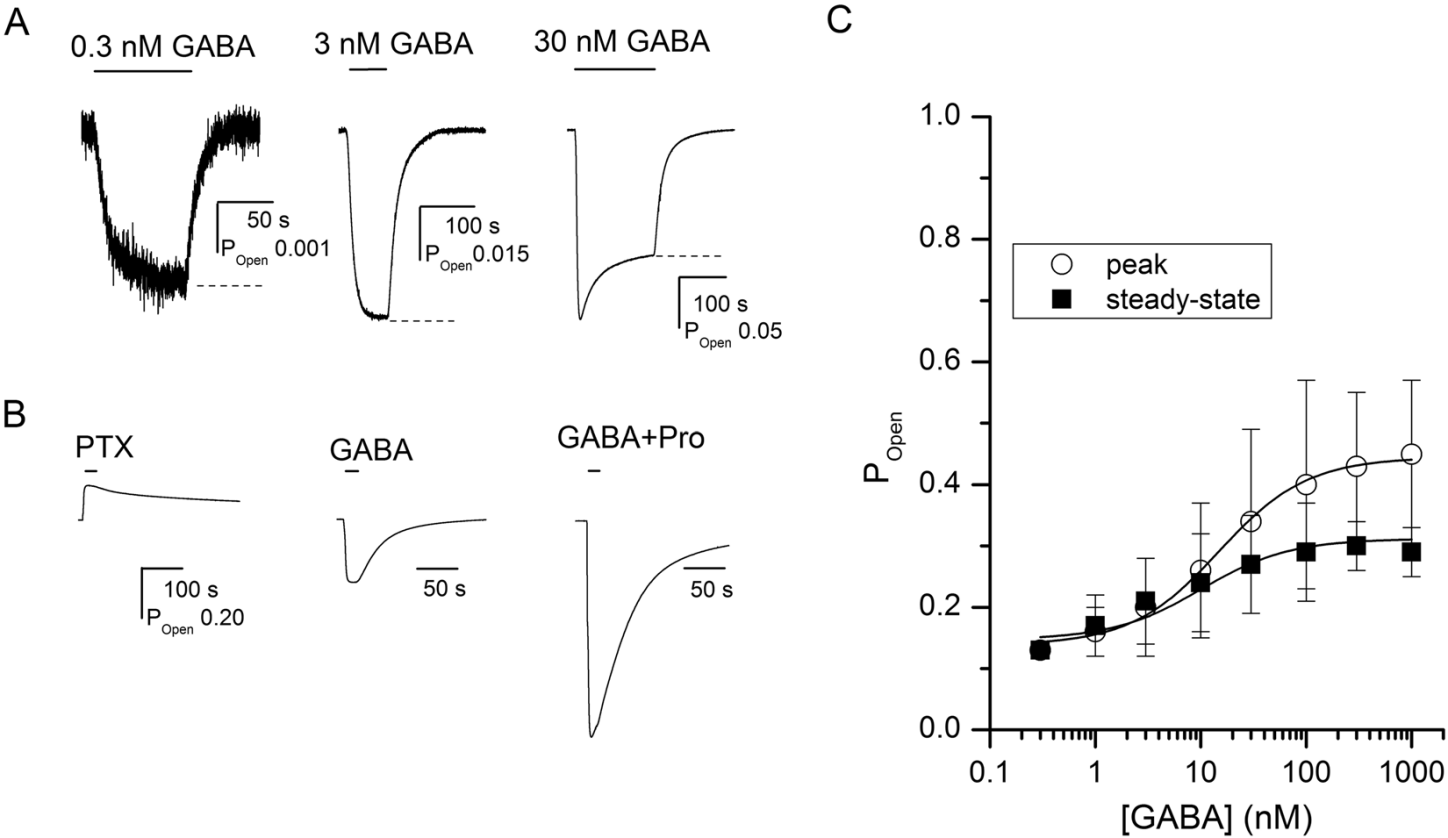
Activation of the α4β2δ receptor by GABA. (**A**) Sample current traces showing activation by 0.3 nM (left), 3 nM (middle), or 30 nM (right) GABA. The dashed lines show the estimated steady-state current levels. (**B**) Sample current traces showing inhibition of constitutively active receptors by 300 μM picrotoxin (PTX; P_Open_ = 0), or activation of resting receptors by 0.3 μM GABA (maximal P_Open,GABA_ = 0.4), or activation of resting receptors by 10 μM GABA + 50 μM propofol (P_Open_ = 1). (**C**) GABA concentration-response relationship. The data points show mean ± S.D. from at least five cells per concentration. The curve for peak currents was fitted using Eq. (1). The best-fit parameters are: K_GABA_ = 15.7 ± 2.3 nM, *c*_GABA_ = 0.45 ± 0.01. The number of GABA binding sites was constrained to 2. The curve for steady-state currents was fitted using Eq. (2) with the K_GABA_ and *c*_GABA_ values constrained to those determined in fitting the peak currents. The best-fit value for Q was 0.78 ± 0.08. Curve-fitting was carried out using Origin v. 7.5 (OriginLab, Northampton, MA) on pooled data.

The oocytes expressing α4β2δ GABA_A_ receptors respond to application of GABA with inward current. Concentration-response measurements carried out in the presence of 0.3–1000 nM GABA yielded an EC_50_ of 20 ± 10 nM and a Hill coefficient of 0.80 ± 0.09 (mean ± S.D.; n = 6 cells). Sample current traces in the presence of GABA are shown in Fig. 2A.

To convert the raw current amplitudes to units of open probability (P_Open_), we compared the response to saturating GABA (0.3 μM) to the response to 300 μM picrotoxin (PTX) and the response to 10 μM GABA + 50 μM propofol. The details of this approach have been reported previously^6,7^. Blockade of activity from constitutively active receptors by PTX is expected to lead to zero GABAergic activity (P_Open_ approaching 0), and receptor activation by the combination of saturating GABA and a high concentration of propofol is expected to generate a maximal possible peak response with a P_Open_ indistinguishable from 1. Comparison of the holding current and peak responses to PTX, GABA, and GABA + propofol, yielded an estimate of 0.13 ± 0.09 (n = 24 cells) for constitutive open probability (P_Open,const_), and an estimate of 0.35 ± 0.09 (n = 22 cells) for open probability in the presence of 0.3 μM GABA. Sample current responses to PTX, GABA, and GABA + propofol are given in Fig. 2B.

The activation parameters for peak responses were determined by fitting the P_Open_ data to Eq. (1)^8,9^:

1 $${{\rm{P}}}_{\mathrm{Open},\mathrm{Peak}}=\frac{{\rm{1}}}{{\rm{1}}+{\rm{L}}{[\frac{{\rm{1}}+[{\rm{X}}]/{{\rm{K}}}_{{\rm{X}}}}{{\rm{1}}+[{\rm{X}}]/({{\rm{K}}}_{{\rm{X}}}{c}_{{\rm{X}}})}]}^{{{\rm{N}}}_{{\rm{X}}}}}$$

where [X] is the concentration of agonist X (GABA in this experiment), K_X_ is the equilibrium dissociation constant for agonist X of the resting receptor, *c*_X_ is the ratio of the equilibrium dissociation constant for X of the open receptor to K_X_, and N_X_ is the number of agonist binding sites. L expresses the level of background activity, and can be calculated from constitutive activity as: (1 − P_Open,const_)/P_Open,const_.

Curve-fitting of pooled data from 6 cells to Eq. (1) yielded a K_GABA_ of 15.7 ± 2.3 nM (best-fit parameter ± S.E. of the fit) and a *c*_GABA_ of 0.45 ± 0.01. The number of GABA binding sites was held at two^10^. The concentration-response relationship for peak currents is given in Fig. 2C.

The data indicate that GABA is a weak agonist of the α4β2δ receptor. The binding of two GABA molecules contributes only 0.94 kcal/mol (${{\rm{N}}}_{{\rm{GABA}}}{\rm{RT}}\times \,\mathrm{ln}({c}_{{\rm{GABA}}})$) towards stabilization of the open state. For comparison, in the synaptic-type α1β2γ2L receptor, the binding of two GABA molecules contributes 6.4–7.5 kcal/mol of stabilization energy^11,12^. Thus, despite the relatively high constitutive open probability (i.e., low intrinsic energy barrier towards channel opening), the theoretical peak maximal open probability of the α4β2δ receptor in the presence of GABA, calculated as $1/(1+{\rm{L}}{{c}_{{\rm{GABA}}}}^{{{\rm{N}}}_{{\rm{GABA}}}})$, is only 0.44. This is in agreement with previous estimates in single-channel and macroscopic studies demonstrating that GABA is a partial agonist of the α4βδ receptor^13–17^.

To analyze the desensitization properties of the α4β2δ receptor, we fitted the concentration-response relationship for steady-state currents to Eq. (2)^5^:

2 $${{\rm{P}}}_{{\rm{Open}},{\rm{S}}.{\rm{S}}.}=\frac{1}{1+\frac{1}{{\rm{Q}}}+{\rm{L}}{[\frac{1+[{\rm{X}}]/{{\rm{K}}}_{{\rm{X}}}}{1+[{\rm{X}}]/({{\rm{K}}}_{{\rm{X}}}{c}_{{\rm{X}}})}]}^{{{\rm{N}}}_{{\rm{X}}}}}$$

The parameter Q (=A/D) reflects the equilibrium between the active and desensitized states (Fig. 1). The other terms are as described for Eq. (1). Curve fitting of steady-state responses, using K_GABA_ and *c*_GABA_ constrained to the values determined for peak currents in the same set of cells, yielded an estimate of 0.78 ± 0.08 for Q. Thus, under steady-state conditions, the ratio of open/active vs. desensitized receptors is ~4:5.

Taurine, an endogenous sulfonic acid and a structural analog of GABA, can activate the GABA_A_ receptor^18–21^. Its effects are likely mediated through interactions with the transmitter binding sites, as suggested by molecular modeling^22^ and the finding that the β2(Y205S) mutation in the transmitter binding site that abolishes receptor activation by GABA^10^ also eliminates activation of the α1β2γ2L and α4β2δ receptors by taurine (<0.2% of the response to GABA + propofol; data not shown).

Taurine concentration-response measurements on oocytes expressing the α4β2δ GABA_A_ receptor yielded an EC_50_ of 9.8 ± 4.8 μM and a Hill coefficient of 0.70 ± 0.08 (n = 6 cells) for peak currents. Fitting the concentration-response data to Eq. (1) gave the estimates of K_taurine_ of 10.0 ± 2.1 μM and a *c*_taurine_ of 0.47 ± 0.02. Thus, taurine and GABA have similar gating efficacies (i.e., *c*_taurine_ ≈ *c*_GABA_) on the α4β2δ receptor and maximal peak open probabilities (0.44 and 0.42, respectively). In recordings in the presence of long (190–410 s) applications of 1 mM taurine, the steady-state open probability was 0.23 ± 0.04 (n = 5 cells), yielding a calculated value of 0.52 for Q.

### Activation and desensitization by the allosteric agonists propofol and 3α5αP

**Figure 3:**
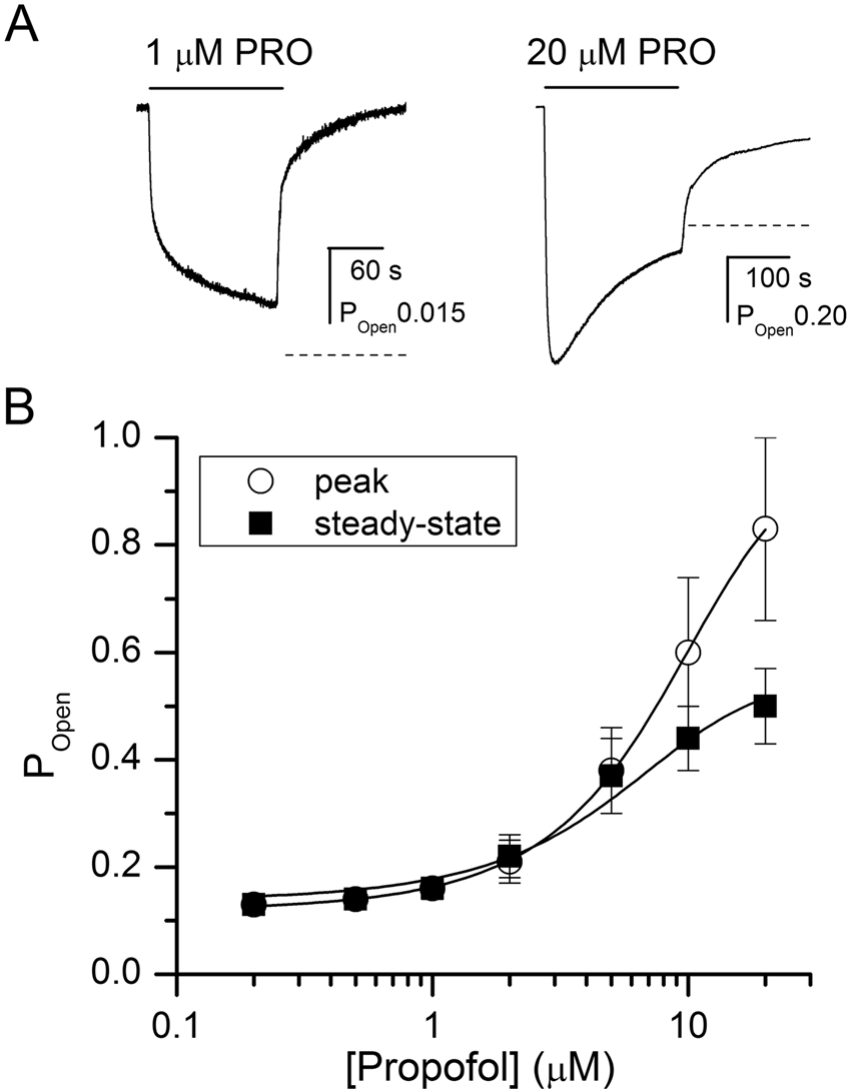
Activation of the α4β2δ receptor by propofol. (**A**) Sample current traces showing activation by 1 μM (left) or 20 μM (right) propofol. The dashed lines show the estimated steady-state current levels. (**B**) Propofol concentration-response relationship. The data points show mean ± S.D. from at least five cells per concentration. The curve for peak currents was fitted using Eq. (1). The best-fit parameters are: K_propofol_ = 55.1 ± 6.6 μM, *c*_propofol_ = 0.16 ± 0.01. The number of propofol binding sites was held at 4. The curve for steady-state currents was fitted using Eq. (2) with the K_propofol_ and *c*_propofol_ values constrained to those determined in fitting the peak currents. The best-fit value for Q was 1.29 ± 0.14. Curve-fitting was carried out using Origin v. 7.5 (OriginLab, Northampton, MA) on pooled data.

The propofol concentration-response relationship was obtained by exposing oocytes expressing the α4β2δ receptor to 0.2–20 μM propofol. Curve-fitting the peak response data with the Hill equation yielded an EC_50_ of 7.3 ± 2.0 μM and a Hill coefficient of 2.17 ± 0.65 (n = 6 cells). Fitting the pooled data to Eq. (1) gave a K_propofol_ of 55.1 ± 6.6 μM and a *c*_propofol_ of 0.16 ± 0.01. The number of binding sites for propofol was constrained to 4. Thus, the binding of propofol to the α4β2δ receptor contributes 4.3 kcal/mol towards stabilization of the open state. The predicted maximal peak P_Open_ in the presence of propofol is ~0.99. Sample current responses and the concentration-response curves are given in Fig. 3.

**Figure 4:**
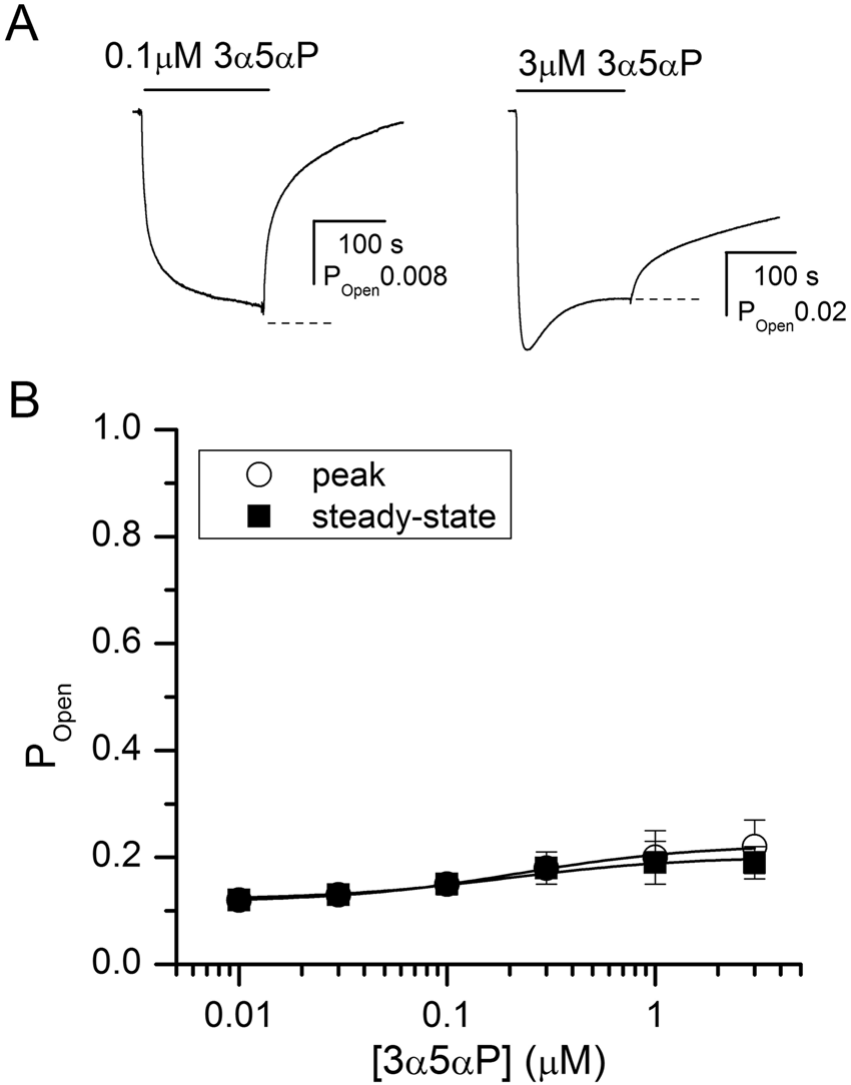
Activation of the α4β2δ receptor by 3α5αP. (**A**) Sample current traces showing activation by 0.1 μM (left) or 3 μM (right) 3α5αP. The dashed lines show the estimated steady-state current levels. (**B**) 3α5αP concentration-response relationship. The data points show mean ± S.D. from at least five cells per concentration. The curve for peak currents was fitted using Eq. (1). The best-fit parameters are: K_3α5αP_ = 0.21 ± 0.04 μM, *c*_3α5αP_ = 0.68 ± 0.01. The number of 3α5αP binding sites was held at 2. The curve for steady-state currents was fitted using Eq. (2) with the K_3α5αP_ and *c*_3α5αP_ values constrained to those determined in fitting the peak currents. The best-fit value for Q was 0.89 ± 0.33. Curve-fitting was carried out using Origin v. 7.5 (OriginLab, Northampton, MA) on pooled data.

Curve-fitting the peak response data recorded in the presence of 0.01–3 μM 3α5αP yielded an EC_50_ of 0.23 ± 0.10 μM and a Hill coefficient of 1.17 ± 0.30 (n = 6 cells). Analysis of the peak currents using Eq. (1) gave a K_3α5αP_ of 0.21 ± 0.04 μM and a *c*_3α5αP_ of 0.68 ± 0.01 with the number of binding sites for 3α5αP held at 2. Sample current responses and the concentration-response relationships are shown in Fig. 4.

To determine receptor desensitization properties in the presence of propofol or 3α5αP, we analyzed the steady-state currents using Eq. (2). With the K and *c* values constrained to the values estimated by analyzing peak responses, we obtained the estimates for Q of 1.29 ± 0.14 in the presence of propofol, and 0.89 ± 0.33 in the presence of 3α5αP. A higher value of Q is associated with reduced desensitization, i.e., a higher steady-state to peak ratio.

We recently showed that propofol enhances steady-state activity elicited by saturating GABA in the α1β2γ2L receptor^23^. The effect, which is observed as an increase in the apparent value of Q, was attributed to propofol having a higher affinity to the open vs. desensitized state. To determine whether an analogous mechanism underlies the higher value of Q in the α4β2δ receptor activated by propofol, we compared the potentiating effect of propofol on peak and steady-state currents elicited by saturating GABA. We reasoned that if propofol potentiates the responses by enhancing receptor open probability then the potentiating effect will be similar for peak and steady-state activity. On the other hand, if propofol additionally reduces receptor desensitization, then the potentiating effect of propofol on steady-state current should exceed that on the peak response. In five cells, coapplication of 1 μM propofol enhanced the peak response to 0.3 μM GABA to 151 ± 12% of control. Application of 1 μM propofol on steady-state response elicited by 0.3 μM GABA augmented the response to 145 ± 14% of control (n = 5 cells). We infer that within the limits of our experimental precision, propofol does not modify the equilibrium between active and desensitized states.

### Modulation of steady-state current by PS

The endogenous steroid PS promotes desensitization of the synaptic-type αβγ GABA_A_ receptor^5,24,25^. Here, we determined the effect of PS on the α4β2δ receptor.

The receptors were activated by a prolonged application of 0.3 μM GABA. Once steady-state response was reached, the flow was switched to GABA + PS. The concentration of PS ranged from 0.1 to 10 μM. Curve-fitting of pooled data from 5–7 cells per concentration gave an IC_50_ of 1.4 ± 0.3 μM and a high concentration asymptote of 50 ± 4% of control.

In the framework of the RAD model, PS inhibits receptor activity by binding with high affinity to the desensitized state and with low affinity to the resting and active states. For receptors activated by GABA, the open probability in the presence of PS is:

3 $${{\rm{P}}}_{{\rm{Open}},{\rm{S}}.{\rm{S}}.}=\frac{1}{1+\frac{1}{{\rm{Q}}}(\frac{1+[{\rm{PS}}]\,/\,({{\rm{K}}}_{{\rm{PS}}}{d}_{{\rm{PS}}})}{1+[{\rm{PS}}]\,/\,{{\rm{K}}}_{{\rm{PS}}}})+{\rm{L}}{[\frac{1+[{\rm{GABA}}]/{{\rm{K}}}_{{\rm{GABA}}}}{1+[{\rm{GABA}}]/({{\rm{K}}}_{{\rm{GABA}}}{c}_{{\rm{GABA}}})}]}^{{{\rm{N}}}_{{\rm{GABA}}}}}$$

where K_PS_ is the equilibrium dissociation constant of the resting and active receptors to PS, and *d*_PS_ is the ratio of the equilibrium dissociation constant of the desensitized receptor to K_PS_. The number of sites for PS was assumed to be 1. Other terms are as described above for Eqs (1,2).

**Figure 5:**
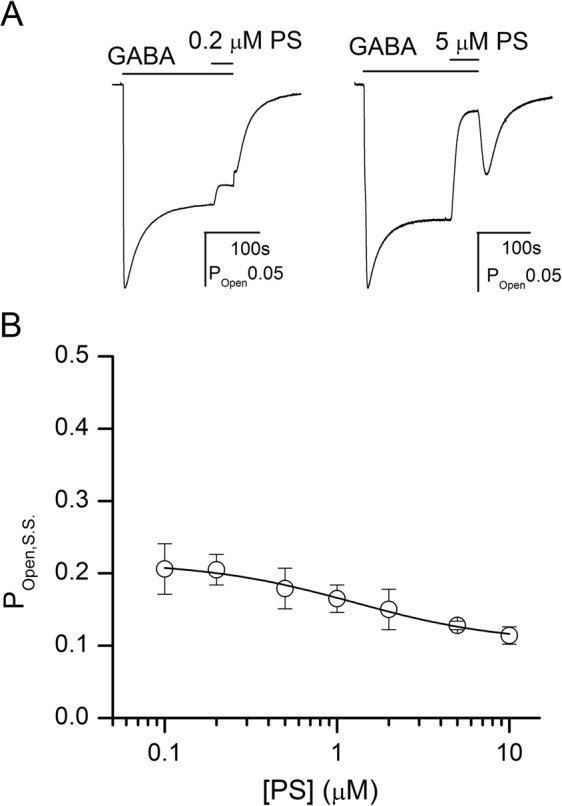
Inhibition of the α4β2δ receptor by pregnenolone sulfate. (**A**) Sample current traces showing the effects of 0.2 μM (left) or 5 μM (right) pregnenolone sulfate (PS) on steady-state current elicited by 0.3 μM GABA. The rebound current following washout of GABA + 5 μM PS likely results from faster washout of PS revealing activity of GABA-bound active receptors. (**B**) PS concentration-response relationship in the presence of 0.3 μM GABA. The data points show mean ± S.D. from at least five cells per concentration. The curve was fitted with Eq. (3), yielding a K_PS_ of 2.6 ± 0.7 μM and a *c*_PS_ of 0.14 ± 0.02. The number of PS binding sites was held at 1. Curve-fitting was carried out using Origin v. 7.5 (OriginLab, Northhampton, MA) on pooled data. The fitted values for K_PS_ and *c*_PS_ are given as best-fit parameter ± standard error of the fit.

In this model, PS does not modify the intrinsic properties of the receptor (i.e., L or Q) or the parameters of receptor activation by GABA (i.e., K_GABA_ or *c*_GABA_). Fitting the PS concentration-response data to Eq. (3) yielded a K_PS_ of 2.6 ± 0.6 μM, and a *d*_PS_ of 0.14 ± 0.02. The K_PS_ and *d*_PS_ estimates are similar to the values previously determined for the α1β2γ2L receptor (1.9 μM and 0.11, respectively)^23^. Sample current traces, the concentration-response data, and the fitted curve are shown in Fig. 5.

### Steady-state activation in the presence of combinations of GABA, taurine, propofol, 3α5αP and/or PS

We previously showed for the α1β2γ2L GABA_A_ receptor that steady-state activity in the presence of multiple active agents is determined by energetic additivity^5,23^. To verify that the same mechanism determines steady-state activity of the α4β2δ receptor, and to gain insight into receptor function in the presence of multiple endogenous and clinical activators and modulators, we measured steady-state responses in the presence of combinations of orthosteric (GABA, taurine) and allosteric activators (propofol, 3α5αP) and inhibitors (PS). The experimentally observed P_Open,S.S._ was compared with the predicted P_Open,S.S._. The latter can be calculated using Eq. (4):

4 $${{\rm{P}}}_{{\rm{Open}},{\rm{S}}.{\rm{S}}.}=\tfrac{1}{1+\tfrac{1}{{\rm{Q}}}(\tfrac{1+[{\rm{PS}}]\,/\,({{\rm{K}}}_{{\rm{PS}}}{d}_{{\rm{PS}}})}{1+[{\rm{PS}}]\,/\,{{\rm{K}}}_{{\rm{PS}}}})+{\rm{L}}{[\tfrac{1+[{\rm{GABA}}]/{{\rm{K}}}_{{\rm{GABA}}}+[{\rm{taurine}}]/{{\rm{K}}}_{{\rm{taurine}}}}{1+[{\rm{GABA}}]/({{\rm{K}}}_{{\rm{GABA}}}{c}_{{\rm{GABA}}})+[{\rm{taurine}}]/({{\rm{K}}}_{{\rm{taurine}}}{c}_{{\rm{taurine}}})}]}^{{{\rm{N}}}_{{\rm{transmitter}}}}{\Gamma }_{[{\rm{propofol}}]}{\Gamma }_{[3\alpha 5\alpha {\rm{P}}]}}$$

where Γ_propofol_ is:

5 $${\Gamma }_{[{\rm{propofol}}]}={[\frac{{\rm{1}}+[{\rm{propofol}}]/{{\rm{K}}}_{{\rm{propofol}}}}{{\rm{1}}+[{\rm{propofol}}]/({{\rm{K}}}_{{\rm{propofol}}}{c}_{{\rm{propofol}}})}]}^{{{\rm{N}}}_{{\rm{propofol}}}}$$

and Γ_3α5αP_ is:

6 $${\Gamma }_{[3{\rm{\alpha }}5{\rm{\alpha }}P]}={[\frac{1+[3{\rm{\alpha }}5{\rm{\alpha }}P]/{{\rm{K}}}_{3{\rm{\alpha }}5{\rm{\alpha }}P}}{1+[3{\rm{\alpha }}5{\rm{\alpha }}P]/({{\rm{K}}}_{3{\rm{\alpha }}5{\rm{\alpha }}P}{c}_{3{\rm{\alpha }}5{\rm{\alpha }}P})}]}^{{{\rm{N}}}_{3{\rm{\alpha }}5{\rm{\alpha }}P}}$$

In practice, however, the predicted P_Open,S.S._ was calculated using Eq. (7):

7 $${{\rm{P}}}_{{\rm{Open}},{\rm{S}}{\rm{.S}}{\rm{.}}}=\frac{1}{1+\frac{1}{{\rm{Q}}}(\frac{1+[{\rm{PS}}]\,/\,({{\rm{K}}}_{{\rm{PS}}}{d}_{{\rm{PS}}})}{1+[{\rm{PS}}]\,/\,{{\rm{K}}}_{{\rm{PS}}}})+{\Pi }_{{\rm{Sum}}}}$$

where Π_Sum_ is a measure of peak activation by the mixture of agonists and is related to the peak open probability as:

8 $${\Pi }_{{\rm{Sum}}}=\frac{1}{{{\rm{P}}}_{{\rm{Open}},{\rm{peak}}}}-1$$

Equations (7) and (8) express steady-state open probability as a dependent product of peak open probability, related to it through Q and the effect of PS (K_PS_ and *d*_PS_) on steady-state current. This approach enabled us to compensate for cell-to-cell variability in the actions of agonist mixtures.

**Figure 6:**
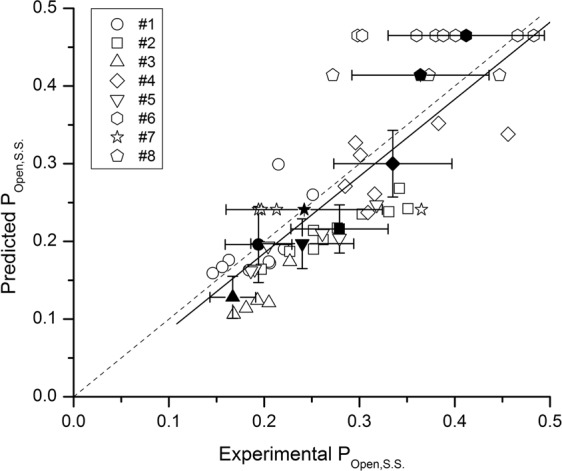
Steady-state activation of the α4β2δ receptor by combinations of orthosteric and allosteric agonists and the inhibitory steroid PS. The graph shows the observed and predicted P_Open_ of steady-state responses in the presence of 100 nM GABA + 10 μM taurine + 30 nM 3α5αP + 0.1 μM PS (drug combination #1), 100 nM GABA + 10 μM taurine + 30 nM 3α5αP + 0.1 μM PS + 1 μM propofol (#2), 10 nM GABA + 10 μM taurine + 10 nM 3α5αP + 1 μM PS (#3), 300 nM GABA + 100 μM taurine + 30 nM 3α5αP + 1 μM propofol (#4), 100 nM GABA + 10 μM taurine + 0.2 μM PS (#5), 10 μM GABA + 50 μM propofol (#6), 10 μM GABA + 50 μM propofol + 1 μM PS (#7), 10 μM GABA + 50 μM propofol + 0.1 μM PS (#8). The predicted P_Open,S.S._ were determined using Eq. (7). The open symbols show data from individual cells. The filled symbols show mean ± S.D. Drug combinations #6–8 contained 10 μM GABA + 50 μM propofol that generates a peak P_Open_ indistinguishable from 1. Accordingly, the S.D. for predicted P_Open,S.S._ are not shown for these combinations. The solid line gives the linear fit to all data points (R^2^ = 0.82, *P* < 0.0001). The dashed line shows ideal agreement between predicted and experimental P_Open,S.S._.

In total, 8 combinations of drugs and drug concentrations were tested. The concentration of GABA ranged from 10 nM to 10 μM, taurine from 10 to 100 μM, propofol from 1 to 50 μM, 3α5αP from 10 to 30 nM, and PS from 0.1 to 1 μM. Not all combinations included all 5 compounds. The data from 53 cells are shown in Fig. 6. A linear fit to all data points yielded an R^2^ of 0.82 (P < 0.0001) with a regression slope of 0.99 ± 0.10.

## Discussion

Receptors consisting of α4, β2 or β3, and δ subunits are a major extrasynaptic type of GABA_A_ receptors in several brain regions such as the hippocampus and the thalamus^26–29^. Prior studies have indicated that the α4βδ receptor has a high affinity to GABA, and is only moderately desensitized during prolonged application of agonist^30–33^. Both properties support its presumed function to mediate tonic Cl^−^ conductance in response to ambient GABA, and the concentration profile of ambient GABA in the brain. The α4βδ receptor is also activated by taurine, endogenous potentiating steroids, and various GABAergic sedative and anesthetic agents^3,14,20,30^. The overall goal of this study was to analyze the function of the α4β2δ receptor in the presence of one or more activators and modulators under steady-state conditions.

Previous work has provided evidence for two types or isoforms of receptors resulting from the expression of α4, β (β2 or β3), and δ subunits. In electrophysiological recordings, this manifests as widely different sensitivities to the agonist. The high-affinity type has a GABA EC_50_ at <100 nM whereas the low-affinity type has a GABA EC_50_ at >1 μM. In some cases, concentration-response relationships show two components in a single cell indicating that both types of the receptor can express concurrently^31^. The underlying reason for differing sensitivity to the agonist is not fully established. Several studies have suggested that it is due to the “promiscuous” nature of the δ subunit that allows for variability in the assembly order of subunits and stoichiometry of the surface receptor. For example, Hartiadi *et al*.^34^ showed that reduction in the ratio of α4 to β2 cRNAs tends to generate receptors with high affinity to GABA whereas changes in δ have no effect. We previously showed that linking individual subunits to concatemeric constructs enables selective generation of low- or high-affinity receptors^32^. In contrast, for receptors activated by the conformationally constrained analog of GABA, THIP, Meera *et al*.^35^ proposed that the two types of behavior are simply due to a mixture of low-affinity αβ and high-affinity αβδ receptors, i.e., incomplete incorporation of δ in all surface receptors. We note that our study was conducted on the “high-affinity” isoform of the α4β2δ receptor.

We have shown that the α4β2δ receptor exhibits relatively high constitutive open probability (P_Open,const_ = 0.1). In the cyclic MWC model, high background activity is associated with enhanced sensitivity to agonist because of a lower energy barrier that needs to be crossed during transition from closed/resting to open/active^9,36^. Despite the high P_Open,const_, the receptor is only weakly activated by the endogenous agonists GABA and taurine. The maximal peak open probabilities were ~0.4 for either agonist. However, both GABA and taurine are relatively potent agonists, and the equilibrium dissociation constants for GABA (~15 nM) and taurine (10 μM) are near their reported extracellular concentrations of 5–30 nM and 10–25 μM, respectively^37–39^. The receptor is weakly directly activated by the endogenous steroid 3α5αP (maximal peak P_Open_ ~0.2), but the intravenous anesthetic propofol is a full agonist (P_Open,max_ ~0.99).

The estimate for Q (=A/D in Fig. 1) was 0.52 in the presence of taurine, 0.78 in the presence of GABA, 0.89 with 3α5αP, and 1.29 when the receptors were activated by propofol. Followup experiments showed that propofol similarly potentiates peak and steady-state currents from receptors activated by GABA. We infer that the observed difference in Q for GABA vs. propofol is a result of experimental imprecision rather than higher affinity of propofol to the open state as previously observed for the α1β2γ2L receptor^23^. In subsequent simulations, we used a value of Q of 0.87, averaged from the individual estimates in the presence of taurine, GABA, 3α5αP, or propofol.

We tested the independence of the actions of orthosteric and allosteric agents by coapplying various combinations of such agents, and comparing the observed P_Open,S.S._ with a predicted value calculated using Eq. (7), which assumes additive effects of each agonist and inhibitor. Overall there was a good agreement between predicted and observed data (Fig. 6). We infer that the actions GABA, taurine, propofol, 3α5αP, and PS on the α4β2δ receptor follow the basic rules of energetic additivity. We did not test energetic additivity of the drugs on peak responses.

The data indicate that taurine is a potent agonist of the α4β2δ receptor with an EC_50_ (10 μM) near its extracellular concentration in the resting state in brain^37^. This is in agreement with a previous study that showed increased tonic current and reduced action potential firing in the presence of 10–100 μM taurine in the thalamus^20^. The reported EC_50_ for taurine on recombinant α4β2δ receptors in HEK cells was, however, higher by several orders of magnitude^20^. We propose that this discrepancy arises from the HEK cells preferentially expressing the low-affinity isoform of the α4β2δ receptor^32^.

Taurine and GABA act additively rather than synergistically because both agonists interact with the same binding site. The calculated (Eq. (4)) steady-state P_Open_ of the α4β2δ receptor is 0.24 in the presence of 30 nM GABA, 0.21 in the presence of 10 μM taurine, and 0.25 in the presence of GABA + taurine. The predicted P_Open,S.S._ in the simultaneous presence of physiological concentrations of major endogenous GABAergic agonists and modulators - 30 nM GABA^39^, 10 μM taurine^37^, 30 nM 3α5αP^40^, and 0.1 μM PS^40^ - is 0.24. The addition of 1 μM propofol^41^ increases the P_Open,S.S._ to 0.28. Such a small potentiating effect may be expected given the low affinity of the receptor for propofol (K_propofol_ > 50 μM). The full extent of physiological significance of these predictions is unclear, but the results tend to argue against the α4β2δ receptor being a significant target for propofol.

The overall predicted theoretical dynamic range of steady-state activity in the α4β2δ receptor is relatively small, ranging from ~0.10 (constitutive activity) to ~0.45 (maximal allowable steady-state activity with Q = 0.87). We speculate that the α4β2δ receptor acts to stabilize the membrane potential near the Cl^−^ reversal potential, and that surface receptor turnover plays a relatively large role in regulation of its function.

It is not fully established which affinity isoform is the best recombinant model of the native, neuronally-expressed extrasynaptic receptor. Several lines of evidence support the idea that the “high-affinity” isoform is a better analog of the native receptor. Submicromolar concentrations of THIP activate tonic current in cerebellar granule cells that is missing in the cells from δ knockout mice^35^. The α4β3δ receptors expressed in oocytes produced THIP concentration-response curves with a high-affinity component at <100 nM (assumed to be analogous to high affinity to GABA) and a low-affinity component at >10 μM^35^. A low concentration (10–100 μM) of taurine elicits tonic inhibitory currents in thalamic neurons^20^. This agrees with our study of the high-affinity isoform in oocytes where we saw strong activation in the 1–100 μM range (see above), but not with concentration-response studies of the α4β2δ receptor expressed in HEK cells^20^, which preferentially express the low-affinity isoform^32^. The physiological relevance of the high-affinity isoform is indirectly supported by the finding that the steroid alfaxalone elicits large currents in the presence of picrotoxin in hippocampal neurons transfected with α4β2δ(T269Y) subunits^32^. Finally, we note that the high-affinity isoform of the α4β2δ receptor with a GABA EC_50_ at 20 nM is expected to be responsive to extracellular (5–30 nM^38,39^) GABA, unlike the low-affinity isoform with an EC_50_ > 1 μM.

## Methods

### Receptors and expression

The human α4β2δ GABA_A_ receptors were expressed in *Xenopus laevis* oocytes. Harvesting of oocytes was conducted under the Guide for the Care and Use of Laboratory Animals as adopted and promulgated by the National Institutes of Health. The animal protocol was approved by the Animal Studies Committee of Washington University in St. Louis (Approval No. 20170071).

The cDNAs of individual subunits in the pcDNA3 vector were linearized with Xba I (NEB Labs, Ipswich, MA). The cRNAs were generated using mMessage mMachine (Ambion, Austin, TX). The oocytes were injected with a total of 11 ng cRNA per oocyte in a 5:1:5 (α4:β2:δ) ratio. Following injection, the oocytes were incubated in bath solution (96 mM NaCl, 2 mM KCl, 1.8 mM CaCl_2_, 1 mM MgCl_2_, and 5 mM HEPES; pH 7.4) plus supplements (2.5 mM Na pyruvate, 100 U/ml penicillin, 100 μg/ml streptomycin, and 50 μg/ml gentamycin) at 15 °C for 3–4 days prior to conducting electrophysiological recordings.

Prior studies have indicated that the α4βδ receptors in oocytes can assemble as isoforms characterized by high affinity to GABA (EC_50_ at tens to hundreds of nM) or low affinity (EC_50_ in the μM range) to GABA^32–35^. The high-affinity isoform has been shown to be directly activated by the δ-specific drug DS-2 whereas the low-affinity isoform is potentiated but not directly activated by DS2^34,42^. The underlying mechanism for this discrepancy is not fully understood, but distinct stoichiometries or subunit order in the two isoforms have been proposed as the cause^32–34^. The isoform investigated in the present study had a high affinity to the transmitter and was directly activated by DS-2.

### Electrophysiology and analysis of current responses

The recordings were conducted at room temperature using standard two-electrode voltage clamp. The pipets were filled with 3 M KCl. The oocytes were clamped at −60 mV. The chamber (RC-1Z, Warner Instruments, Hamden, CT) was perfused with bath solution (see above) at 5–8 ml/min. Solutions were gravity-applied from 30-ml glass syringes with glass luer slips via Teflon tubing, and switched manually.

The current responses were amplified with an Axoclamp 900A (Molecular Devices, Sunnyvale, CA) or OC-725C amplifier (Warner Instruments, Hamden, CT), digitized with a Digidata 1320 or 1200 series digitizer (Molecular Devices), and stored using pClamp (Molecular Devices).

A typical experiment entailed recording of baseline current for 10–20 s, followed by application of a test compound or a combination of compounds for 60–270 s (1–4.5 min), and by application of bath solution to demonstrate recovery. Due to long exposure times, not all cells yielded a full range of concentration-response data. Thus, the concentration-response relationships shown may reflect mean responses from cells exposed to an incomplete range of agonist concentrations. In such cases, the number of cell provided is given as a range of cell numbers for each concentration point. The effects of the inhibitory steroid PS were determined by coapplying the steroid with 0.3 μM GABA. Each cell was tested with 1–3 concentrations of PS. Each cell was also tested with 10 μM GABA + 50 μM propofol to determine the maximal attainable peak response, which was assigned a P_Open_ of 1, and to which the responses to test drugs were compared. This approach assumes that peak responses are not affected by desensitization, i.e., that desensitization develops slowly compared to activation, and that the combination of GABA + propofol activates all resting receptors. The level of constitutive activity was determined by exposing the cells to 100–300 μM picrotoxin.

The current traces were analyzed using Clampfit (Molecular Devices) to determine the amplitudes of peak and steady-state responses. If steady-state (defined as ΔI < 2% during the last 20 s of agonist application) was not reached by the end of the agonist application, an estimate was made by exponential fitting of the current decay. Fitting was done using pClamp, to a single exponential or sums of up to three exponentials. The constant offset is reported as the steady-state response. The estimated value of the offset was relatively insensitive to the number of exponentials used in fitting (up to ~10% variability in the fitted offset).

### Materials and chemicals

The salts and HEPES used to prepare the bath solution, GABA, and 3α5αP were purchased from Sigma-Aldrich (St. Louis, MO). Propofol was purchased from MP Biomedicals (Solon, OH). Pregnenolone sulfate (PS) was bought from Tocris (Bio-Techne, Minneapolis, MN).

The stock solution of GABA was made in the bath solution at 500 mM, stored in aliquots at −20 °C, and diluted on the day of experiment. Stock solution of propofol was made in DMSO at 200 mM and stored at room temperature. 3α5αP was dissolved in DMSO at 10–20 mM and stored at room temperature. PS was dissolved in DMSO at 50 mM and stored at 4 °C.

## Data Availability

The datasets generated and/or analyzed during the current study are available from the corresponding author on reasonable request.

## References

[1] Franks NP (2006). Molecular targets underlying general anaesthesia. Br J Pharmacol.

[2] Weir CJ, Mitchell SJ, Lambert JJ (2017). Role of GABA_A_ receptor subtypes in the behavioural effects of intravenous general anaesthetics. Br J Anaesth.

[3] Liao Y, Liu X, Jounaidi Y, Forman SA, Feng HJ (2019). Etomidate effects on desensitization and deactivation of α4β3δ GABA_A_ receptors inducibly expressed in HEK293 TetR cells. J Pharmacol Exp Ther.

[4] Tang X, Hernandez CC, Macdonald RL (2010). Modulation of spontaneous and GABA-evoked tonic α4β3δ and α4β3γ2L GABA_A_ receptor currents by protein kinase A. J Neurophysiol.

[5] Germann AL, Pierce SR, Burbridge AB, Steinbach JH, Akk G (2019). Steady-state activation and modulation of the concatemeric α1β2γ2L GABA_A_ Receptor. Mol Pharmacol.

[6] Eaton MM (2016). Multiple non-equivalent interfaces mediate direct activation of GABA_A_ receptors by propofol. Curr Neuropharmacol.

[7] Forman SA, Stewart D (2012). Mutations in the GABA_A_ receptor that mimic the allosteric ligand etomidate. Methods Mol Biol.

[8] Akk G, Shin DJ, Germann AL, Steinbach JH (2018). GABA type A receptor activation in the allosteric coagonist model framework: relationship between EC_50_ and basal activity. Mol Pharmacol.

[9] Forman SA (2012). Monod-Wyman-Changeux allosteric mechanisms of action and the pharmacology of etomidate. Curr Opin Anaesthesiol.

[10] Amin J, Weiss DS (1993). GABA_A_ receptor needs two homologous domains of the β-subunit for activation by GABA but not by pentobarbital. Nature.

[11] Ruesch D, Neumann E, Wulf H, Forman SA (2012). An allosteric coagonist model for propofol effects on α1β2γ2L γ-aminobutyric acid type A receptors. Anesthesiology.

[12] Shin DJ, Germann AL, Steinbach JH, Akk G (2017). The actions of drug combinations on the GABA_A_ receptor manifest as curvilinear isoboles of additivity. Mol Pharmacol.

[13] Meera P, Olsen RW, Otis TS, Wallner M (2009). Etomidate, propofol and the neurosteroid THDOC increase the GABA efficacy of recombinant α4β3δ and α4β3 GABA_A_ receptors expressed in HEK cells. Neuropharmacology.

[14] Akk G, Bracamontes J, Steinbach JH (2004). Activation of GABA_A_ receptors containing the α4 subunit by GABA and pentobarbital. J Physiol.

[15] Storustovu SI, Ebert B (2006). Pharmacological characterization of agonists at δ-containing GABA_A_ receptors: Functional selectivity for extrasynaptic receptors is dependent on the absence of γ2. J Pharmacol Exp Ther.

[16] Mortensen, M., Ebert, B., Wafford, K. & Smart, T. G. Distinct activities of GABA agonists at synaptic- and extrasynaptic-type GABA_A_ receptors. *J Physiol* **588**, 1251–1268. 10.1113/jphysiol.2009.182444 PMC2872731 20176630

[17] Keramidas A, Harrison NL (2008). Agonist-dependent single channel current and gating in α4β2δ and α1β2γ2S GABA_A_ receptors. J Gen Physiol.

[18] Horikoshi T, Asanuma A, Yanagisawa K, Anzai K, Goto S (1988). Taurine and β-alanine act on both GABA and glycine receptors in Xenopus oocyte injected with mouse brain messenger RNA. Brain Res.

[19] Hadley SH, Amin J (2007). Rat α6β2δ GABA_A_ receptors exhibit two distinct and separable agonist affinities. J Physiol.

[20] Jia F (2008). Taurine is a potent activator of extrasynaptic GABA_A_ receptors in the thalamus. J Neurosci.

[21] Kletke O, Gisselmann G, May A, Hatt H, Sergeeva OA (2013). Partial agonism of taurine at γ-containing native and recombinant GABA_A_ receptors. PLoS One.

[22] Ochoa-de la Paz LD (2018). Differential modulation of human GABAC-ρ1 receptor by sulfur-containing compounds structurally related to taurine. BMC Neurosci.

[23] Germann, A. L. *et al*. Steady-state activation and modulation of the synaptic-type α1β2γ2L GABA_A_ receptor by combinations of physiological and clinical ligands. *Physiol Rep* **7**, e14230 (2019). 10.14814/phy2.14230 PMC6757177 31549483

[24] Akk G, Bracamontes J, Steinbach JH (2001). Pregnenolone sulfate block of GABA_A_ receptors: mechanism and involvement of a residue in the M2 region of the α subunit. J Physiol.

[25] Eisenman LN, He Y, Fields C, Zorumski CF, Mennerick S (2003). Activation-dependent properties of pregnenolone sulfate inhibition of GABA_A_ receptor-mediated current. J Physiol.

[26] Chandra D (2006). GABA_A_ receptor α4 subunits mediate extrasynaptic inhibition in thalamus and dentate gyrus and the action of gaboxadol. Proc Natl Acad Sci USA.

[27] Porcello DM, Huntsman MM, Mihalek RM, Homanics GE, Huguenard JR (2003). Intact synaptic GABAergic inhibition and altered neurosteroid modulation of thalamic relay neurons in mice lacking δ subunit. J Neurophysiol.

[28] Wisden W, Laurie DJ, Monyer H, Seeburg PH (1992). The distribution of 13 GABA_A_ receptor subunit mRNAs in the rat brain. I. Telencephalon, diencephalon, mesencephalon. J Neurosci.

[29] Stell BM, Brickley SG, Tang CY, Farrant M, Mody I (2003). Neuroactive steroids reduce neuronal excitability by selectively enhancing tonic inhibition mediated by δ subunit-containing GABA_A_ receptors. Proc Natl Acad Sci USA.

[30] Brown N, Kerby J, Bonnert TP, Whiting PJ, Wafford KA (2002). Pharmacological characterization of a novel cell line expressing human α4β3δ GABA_A_ receptors. Br J Pharmacol.

[31] Karim N (2012). Low nanomolar GABA effects at extrasynaptic α4β1/β3δ GABA_A_ receptor subtypes indicate a different binding mode for GABA at these receptors. Biochem Pharmacol.

[32] Eaton MM (2014). γ-aminobutyric acid type A α4, β2, and δ subunits assemble to produce more than one functionally distinct receptor type. Mol Pharmacol.

[33] Wongsamitkul N, Baur R, Sigel E (2016). Toward understanding functional properties and subunit arrangement of α4β2δ γ-aminobutyric acid, type A (GABA_A_) Receptors. J Biol Chem.

[34] Hartiadi LY, Ahring PK, Chebib M, Absalom NL (2016). High and low GABA sensitivity α4β2δ GABA_A_ receptors are expressed in *Xenopus laevis* oocytes with divergent stoichiometries. Biochem Pharmacol.

[35] Meera, P., Wallner, M. & Otis, T. S. Molecular basis for the high THIP/gaboxadol sensitivity of extrasynaptic GABA_A_ receptors. *J Neurophysiol* (2011). 10.1152/jn.00450.2011 PMC3191842 21795619

[36] Steinbach JH, Akk G (2019). Applying the Monod-Wyman-Changeux allosteric activation model to pseudo-steady-state responses from GABA_A_ receptors. Mol Pharmacol.

[37] Molchanova S, Oja SS, Saransaari P (2004). Characteristics of basal taurine release in the rat striatum measured by microdialysis. Amino Acids.

[38] de Groote L, Linthorst AC (2007). Exposure to novelty and forced swimming evoke stressor-dependent changes in extracellular GABA in the rat hippocampus. Neuroscience.

[39] Zandy SL, Doherty JM, Wibisono ND, Gonzales RA (2017). High sensitivity HPLC method for analysis of *in vivo* extracellular GABA using optimized fluorescence parameters for *o*-phthalaldehyde (OPA)/sulfite derivatives. J Chromatogr B Analyt Technol Biomed Life Sci.

[40] Weill-Engerer S (2002). Neurosteroid quantification in human brain regions: comparison between Alzheimer’s and nondemented patients. J Clin Endocrinol Metab.

[41] Dawidowicz AL, Kalitynski R, Fijalkowska A (2003). Free and bound propofol concentrations in human cerebrospinal fluid. Br J Clin Pharmacol.

[42] Wafford KA (2009). Novel compounds selectively enhance δ subunit containing GABA_A_ receptors and increase tonic currents in thalamus. Neuropharmacology.

